# Molecular Confirmation of *Taenia crassiceps* Cysticercosis in a Captive Ring-Tailed Lemur (*Lemur catta*) in Poland

**DOI:** 10.3390/pathogens11080835

**Published:** 2022-07-26

**Authors:** Małgorzata Samorek-Pieróg, Jacek Karamon, Adam Brzana, Lesław Sobieraj, Mariusz Włodarczyk, Jacek Sroka, Aneta Bełcik, Weronika Korpysa-Dzirba, Tomasz Cencek

**Affiliations:** 1 Department of Parasitology and Invasive Diseases, National Veterinary Research Institute, Partyzantów Avenue 57, 24-100 Puławy, Poland; j.karamon@piwet.pulawy.pl (J.K.); jacek.sroka@piwet.pulawy.pl (J.S.); aneta.belcik@piwet.pulawy.pl (A.B.); weronika.korpysa@piwet.pulawy.pl (W.K.-D.); tcencek@piwet.pulawy.pl (T.C.); 2 Regional Veterinary Laboratory, Wrocławska 170, 46-020 Opole, Poland; a.brzana@wiw.opole.pl; 3 Zoo Opole, Spacerowa 10, 45-094 Opole, Poland; sobieraj@zoo.opole.pl (L.S.); mariuszw@zoo.opole.pl (M.W.)

**Keywords:** *Taenia crassiceps*, *Cysticercus longicollis*, ring-tailed lemur, nonhuman primate, PCR, Poland

## Abstract

(1) Background: *Taenia crassiceps* is a cosmopolitan tapeworm endemic to the northern hemisphere with an indirect lifecycle. Its definitive hosts are carnivores, and its intermediate hosts are rodents and rabbits. Nonhuman primates in zoos appear to be highly susceptible to *T. crassiceps* cysticercosis. The aim of this study was to confirm the presence and the molecular characterization of *T. crassiceps* cysts isolated from a captive ring-tailed lemur. (2) Methods: Surgery revealed multifocal, transparent saccules containing several thin-walled tapeworm cysticerci. In some of the metacestodes, single or multiple exogenous buds from daughter cysticerci were spotted. A molecular analysis was performed to confirm our morphological examinations, using two protocols to obtain the partial *nad1* and *cox1* genes of the *Taenia* sp. (3) Results: On the basis of morphological features and molecular analysis, the cysticerci were identified as *T. crassiceps* metacestodes, and products taken from the PCRs were sequenced. With respect to interpreting the sequencing results of the obtained amplicons, we compared them with data in the GenBank database, proving that, in this case, the causative agent was indeed *T. crassiceps*. (4) Conclusions: The received data can be used to supplement descriptions of this species. To the best of our knowledge, this is the first case of cysticercosis caused by *T. crassiceps* in a nonhuman primate in Poland.

## 1. Introduction

*Taenia crassiceps* (Zeder, 1800) is a cosmopolitan tapeworm endemic to the northern hemisphere, especially throughout North America, Asia and Europe [1]. It belongs to the Taenidae family and has an indirect lifecycle [2]. Its definitive hosts are carnivores, mainly red foxes (*Vulpes vulpes*), arctic foxes (*Vulpes lagopus*), wolves (*Canis lupus*) and other canids, which harbor the adult tapeworms in their small intestines. Mature parasites can reach up to 20 cm in length. The scolex is spherical and contains four oval suction pads, each 20 µm in diameter. The anterior excrescence is armed with 28–30 hooks arranged in a double crown [2]. Gravid proglottids are excreted with the host’s feces, thus releasing eggs into the environment. Intermediate hosts become infected by ingesting these eggs, which then develop into the larval stages called *Cysticercus longicollis* (metacestodes, cysticerci) [3,4]. It has also been proven that the eggs of *T. crassiceps* can occasionally infect small injuries, hatching within them and then developing locally into the larval stages [5]. The metacestodes are transparent, oval follicles of 4–5 mm in diameter. A single scolex is invaginated or evaginated at one of the poles of the cysticercus. The scolex is equipped with 16–17 hooks and 4 suction pads [2]. At the opposite pole of the scolex, there are buds, which proliferate via asexual budding and produce strings of follicles. *T. crassiceps*, unlike other tapeworms, has the ability to bud exogenously within the host and create additional infective cysticerci outside of a cyst’s bladder. Such bud proliferation can be in the realm of thousands, causing a decline in the health of the host and even death [1]. The species’ natural intermediate hosts are rodents and rabbits, and it tends to harbor itself in their body cavities, subcutaneous tissues, muscles or nervous systems as cyst-like larvae. After eating an infected intermediate host, the cysticerci develop into their adult stages in the small intestine of the definitive host [2]. Noteworthily, animals serving as definitive hosts can also suffer from cysticercosis by *C. longicollis,* which was reported in several studies [4,6,7,8,9]. A variety of routes of exposure resulting in cysticercosis have been postulated, such as autoinfection via eggs from the host’s feces, or eggs from the environment. Nevertheless, the mechanisms by which cysticercosis occurs instead of a patent infection are still unexplained. It is believed that a compromised immune status may favor this infection [7].

Foxes, as the definitive hosts, play a crucial role in the transmission of *T. crassiceps*. In Europe, the observed prevalence of this parasite in red foxes is 17.7–28.5% in Germany [10,11,12], 7.6% in Switzerland [13], 4.3–23% in Spain [14] and 2.7% in northern Italy [15]. Intestinal infections have also been observed in wolves in Latvia (a prevalence of about 8,8%) [16], in stone martens in Germany (a prevalence of about 6%) [10] and raccoon dogs in Lithuania (a prevalence of about 3.5%) [17]. Cases of *T. crassiceps* infection also occur in companion animals (such as dogs); fortunately, this is relatively rare, and in most cases, it is described in connection with the detection of the other parasites. The prevalence of this parasite in dog fecal samples in eastern France is 0.6% [18] and 0.7% in Poland [19]. Of note, it is assumed that close contact between people and pet dogs may favor the infection of humans [20].

Primates can serve as dead-end intermediate hosts after the consumption of water or food contaminated with the eggs of *T. crassiceps*, typically excreted along with carnivore feces [21,22]. The epidemiology of this rare zoonotic disease has not been well investigated. However, this parasite has been used in numerous studies as in an animal model for human cysticercosis, and the characterization of its genome will contribute to the understanding of the human infection [23]. Seemingly low incidences of infection in primates (including people) may be associated with a very low infection pressure or with a relatively high resistance against this particular taeniid species [5]. Moreover, when it comes to people, most cases of the disease have been observed in immunocompromised and HIV-positive patients, e.g., in Germany [22,24], France [25,26], Switzerland [27,28] and the USA [29]. In all of the aforementioned cases, the most common expressions of the disease were the subcutaneous and muscle forms of cysticercosis. While intraocular infections do not appear to require an impaired immune system [30], the larvae of *T. crassiceps* have rarely been discovered in immunocompetent people [22].

Nonhuman primates in zoos appear to be highly susceptible to *T. crassiceps* cysticercosis, which may be a sign of the zoonotic potential of other cestode parasite species [21]. Indeed, several cases of infection in zoo primates have been reported [31,32,33,34,35].

In this article, we describe cysticercosis caused by *T. crassiceps* in a ring-tailed lemur (*Lemur catta*), together with the molecular characterization of a *T. crassiceps* cyst isolated from this case. This is the first confirmed case of this parasite in a nonhuman primate in Poland.

## 2. Results

**Figure 1:**
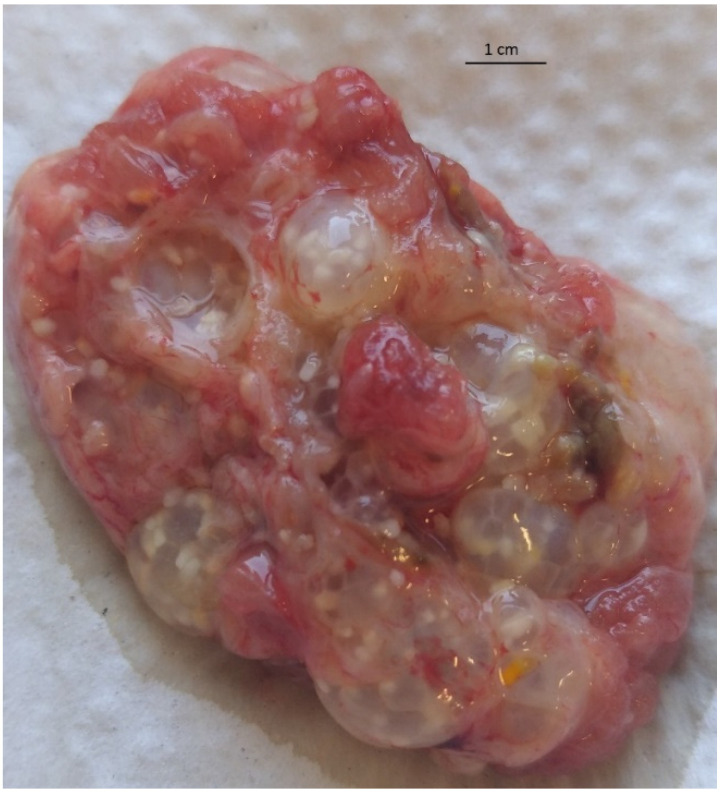
A lesion consisting of multifocal, transparent saccules containing the tapeworm cysticerci removed from the area of the broad fascia tensioner muscle and the biceps femoris muscle of a ring-tailed lemur.

**Figure 2:**
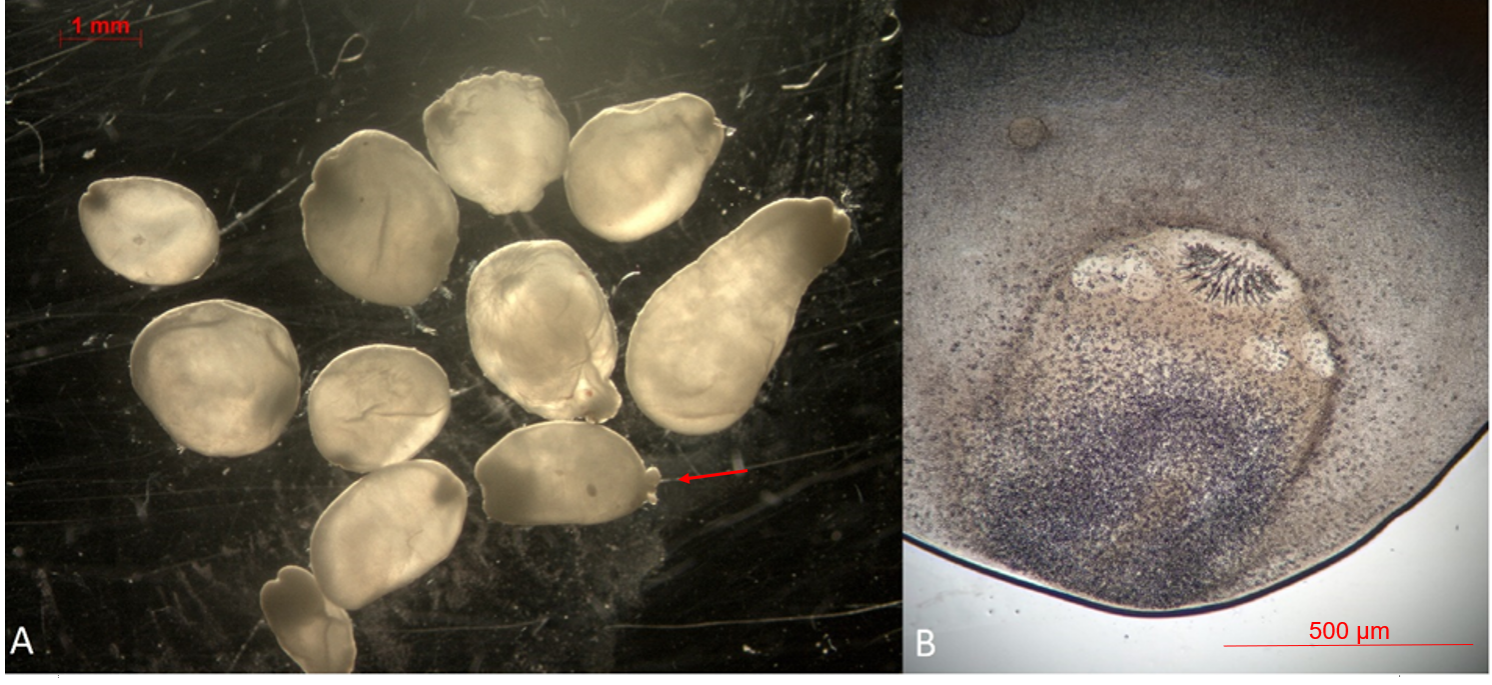
*T. crassiceps* cysts collected from a lesion on a ring-tailed lemur. The exogenous budding was marked with an arrow (**A**). A metacestode of *T. crassiceps* with a single evaginated scolex and a rostellum with two rows of hooks (**B**).

A parasitological examination of the material collected during the cyst removal procedure was performed at the Regional Veterinary Laboratory in Opole. It revealed multifocal, transparent saccules containing several thin-walled tapeworm cysticerci (Figure 1). The metacestodes were round to oval in shape, 1–4 mm in diameter and white to pale yellow in color (Figure 2A). Each cysticercus contained a single evagineted scolex, one or more suckers, a rostellum with two rows of hooks and numerous calcareous corpuscles (Figure 2B). In some of the metacestodes, single or multiple exogenous buds from daughter cysticerci were spotted. The parasite was microscopically identified by its measurements and shape and then compared to the description provided by Loos-Frank [2], which accurately matched it with the description of the *T. crassiceps* metacestode stage.

The molecular analysis confirmed the morphological examinations, and the amplification of the partial sequences of *nad1* and *cox1* was successful. The products of both PCR reactions were the expected sizes. Using the GenBank database, a comparison of the sequencing results from the obtained amplicons confirmed that the detected parasite was *T. crassiceps*.

**Figure 3:**
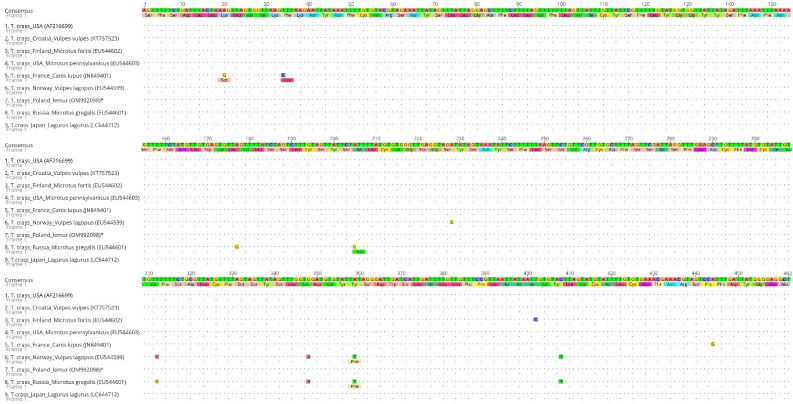
An alignment of the partial *nad1* sequences of *T. crassiceps* (nucleotide sequences together with amino acid sequences) available in the GenBank database with our sequence (* denotes sequence from this study).

A PCR of the *nad1* gene fragment resulted in an amplification product of 488 base pairs (bp) in length, which covers 54.4% (488 bp/897 bp) of the whole gene length. This was added to the GenBank database under the OM992098 accession number. Our sequence was compared to the consensus sequence, created from sequences available in the GenBank database (AF216699.1; KT757523.1; EU544602.1; EU544603.1; JN849401.1; EU544599.1; EU544601.1; LC644712.1). The alignment of our partial *nad1* sequence with the consensus sequence and the sequences from the USA (accession number AF216699.1 and EU544603.1), Croatia (accession number KT757523.1) and Japan (accession number LC644712.1) revealed a 100% identity among them. The G + C content of the analyzed *nad1* fragment was at a 27.3% level. The average base composition of the partial *nad1* gene was A (22.3%), T (50.4%), G (21.4%) and C (5.8%). A translation of the partial *nad1* sequence into an open reading frame resulted in a protein sequence of 154 amino acids in length (Figure 3).

**Figure 4:**
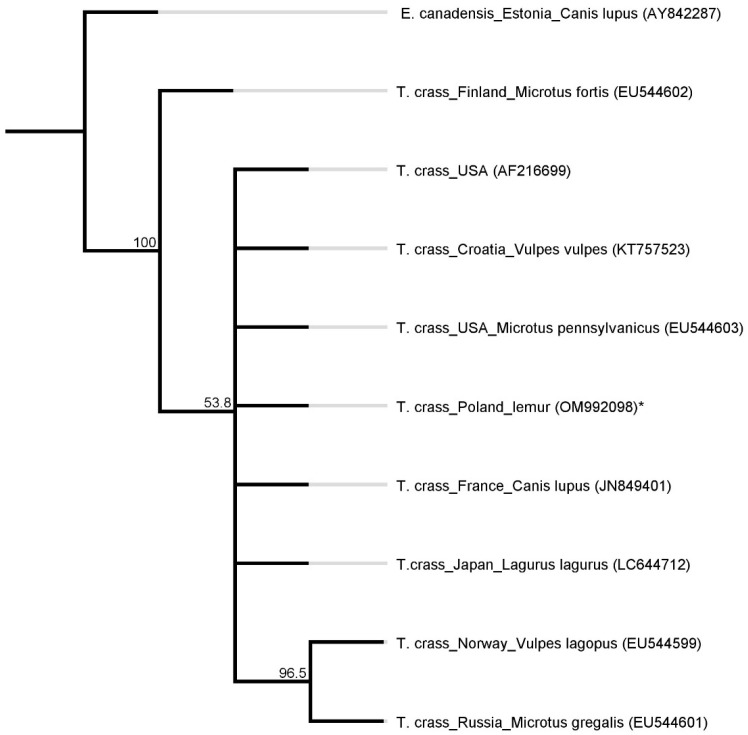
A phylogenetic tree based on a fragment of the *nad1* gene. *T. crass.*—*Taenia crassiceps*; *—isolate from this study. *Echinococcus canadensis* as an outgroup. The values on the tree nodes are bootstrap proportions (%).

A phylogenetic analysis of the abovementioned partial *nad1* sequences showed very little variation. The identity ranged from 98.7 to 100%. According to a phenogram (presented in Figure 4), the detected *T. crassiceps* (* our sequence) genotype belongs to the same clade as *T. crassiceps* sequences isolated from: *Microtus pensylvanicus* from the USA (EU544603.1), *Vulpes vulpes* from Croatia (KT757523.1), *Canis lupus* from France (JN849401.1), *Lagurus lagurus* from Japan (LC644712.1) and another sequence from the USA (AF216699.1).

**Figure 5:**
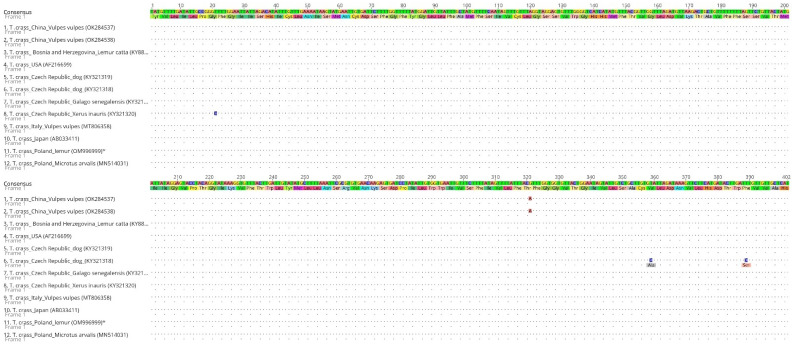
An alignment of the partial *cox1* sequences of *T. crassiceps* (nucleotide sequences together with amino acid sequences) available in the GenBank database with our sequence (* denotes sequence from this study).

A PCR of the *cox1* gene fragment resulted in an amplification product of 446 bp in length, which covers 27.5% (446 bp/1620 bp) of the whole gene length. This was added to the GenBank database under the OM996999 accession number. Our sequence was compared to the consensus sequence created from the sequences available in the GenBank database (sequences that met the criterion of a length greater than 400 bp, and those with determined nucleotides were selected for the analysis) (OK284537.1; OK284538.1; KY883633.1; AF216699.1; KY321319.1; KY321318.1; KY321321.1; KY321320.1; MT806358.1; AB033411.1; MN514031.1). The alignment of our partial *cox1* sequence with the consensus sequence and the sequences from Bosnia and Herzegovina (accession number KY883633.1), the USA (accession number AF216699.1), the Czech Republic (accession number KY321319.1 and KY321321.1), Italy (accession number MT806358.1), Japan (accession number AB033411.1) and Poland (accession number MN514031.1) revealed a 100% identity among them. The G + C content of the analyzed *cox1* fragment was at a 30.8% level. The average base composition of the partial *cox1* gene was A (22.4%), T (46.8%), G (22.6%) and C (8.2%). A translation of the partial *cox1* sequence to an open reading frame resulted in a protein sequence of 148 amino acids in length (Figure 5).

**Figure 6:**
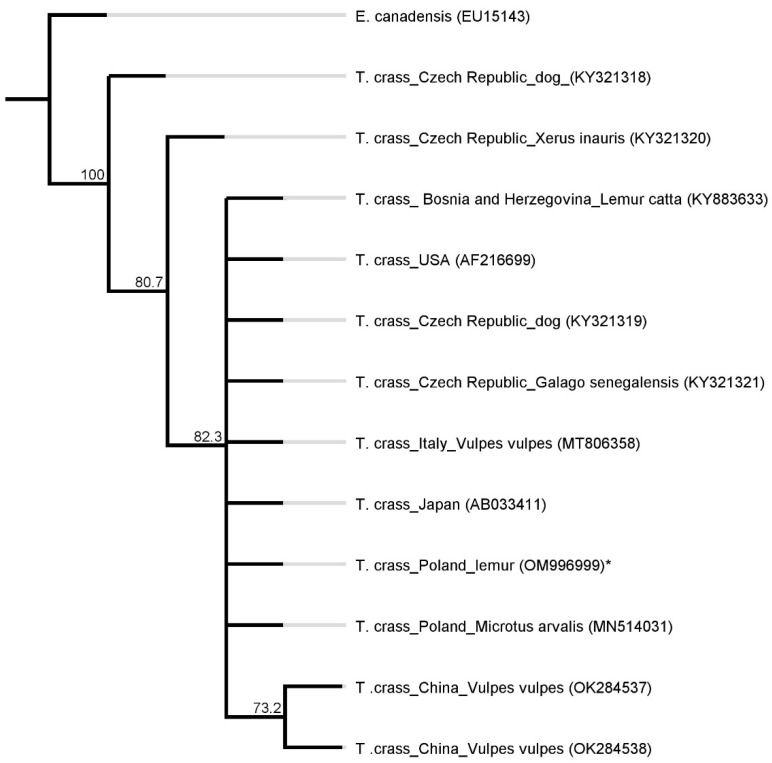
A phylogenetic tree based on a fragment of the *cox1* gene. *T. crass.—Taenia crassiceps*; *—isolate from this study. *Echinococcus canadensis* as an outgroup. The values on the tree nodes are bootstrap proportions (%).

A phylogenetic analysis of the abovementioned partial *cox1* sequences showed very little variation. The identity ranged from 99.5 to 100%. According to a phenogram (presented in Figure 6), the detected *T. crassiceps* (*our sequence) genotype belongs to the same clade as the *T. crassiceps* sequences isolated from: *Lemur catta* from Bosnia and Herzegovina (KY883633.1), *Canis lupus familiaris* from the Czech Republic (KY321319.1), *Galago senegalensis* from the Czech Republic (KY321321.1), *Vulpes vulpes* from Italy (MT806358.1), *Microtus arvalis* from Poland (MN514031.1) and sequences from the USA (AF216699.1) and Japan (AB033411.1).

## 3. Discussion

Despite the fact that the larval stages of *T. crassiceps* are predominantly found in rodents or rabbits [36,37], cases of cysticercosis due to this tapeworm have also been found in many unusual hosts, such as a Cape fur seal [38], a chinchilla [3], a Nigri langur [32] and various lemur species [31,33,34,35]. In this article, a case of subcutaneous cysticercosis in a ring-tailed lemur from a zoo in the city of Opole is described. To the best of our knowledge, this is the first study providing a molecular confirmation of the presence of *T. crassiceps* in a nonhuman primate—that is, a lemur in Poland.

Foxes and dogs, as definitive hosts, play a crucial role in the transmission of *T. crassiceps*; therefore, evidence of a host coming into contact with a tapeworm-carrying animal provides additional epidemiological support for suspecting *T. crassiceps* infection [1]. In our case, the most likely source of infection and mode of transmission to this lemur was the presence of foxes in the area, as well as the occasional, accidental crossing of the natural barriers that separate them from the lemur enclosure. As a consequence, access to feces from a definitive host would have been possible. Our results are in line with those of other researchers [32,34], who found a positive relationship between access to fecal material from red foxes and *T. crassiceps* cysticercosis. The contamination of soil and food (fruits and vegetables) with *T. crassiceps* eggs might also be the source of infection for captive animals. This was confirmed, by studies conducted at the Basel Zoo, Switzerland [39] and the Sarajevo Zoo, Bosnia and Herzegovina [31]. Nevertheless, in the case of our lemur from the Opole Zoo, the possibility of contamination from feed was excluded. Nourishment for the animals, especially for the primates, undergoes strict and specific quality procedures before being released for feeding by the so-called central kitchen.

Reports of *T. crassiceps* cysticercosis in lemurs are rare. Nonetheless, the lesions observed in other animals [35] are consistent with the case of the lemur described in this article. In our case, surgical excision followed by medical therapy was successful, but in some cases, e.g., due to the localization of lesions and an intensive proliferation of cysts, *T. crassiceps* infection can lead to death. Among captive animals, two fatal cases have been reported in ring-tailed lemurs (*Lemur catta*) at the Madrid Zoo in Spain [34] and the Sarajevo Zoo in Bosnia and Herzegovina [31].

In cases of cysticercosis, molecularly confirming the source of infection should be obligatory in order to precisely determine the causative agent. Based on the morphological criteria of the cysticerci, a preliminary diagnosis is possible, but often problematic, due to the great superficial similarity of several morphological characteristics among *Taenia* species. Consequently, combined approaches using well-defined morphological characteristics in conjunction with molecular confirmation should be the standard [20]. In our investigation, a molecular identification with PCR and a sequence analysis were performed to confirm the preliminary diagnosis of *T. crassiceps* infection and the species affiliation.

The occurrence of *T. crassiceps* in Poland has been substantiated in a limited number of studies. The first investigation described this parasite as originating from rodents (*Microtus arvalis*) and red foxes (*Vulpes vulpes*) [37]. The second study revealed the occurrence of *T. crassiceps* in dogs, but only in connection with the detection of other parasites [19]. In the present study, two molecular markers (*nad1* and *cox1*) recovered from a nonhuman primate (*Lemur catta*)—i.e., a dead-end intermediate host—were used to identify the cestode species. These mitochondrial genes can be used to determine intraregional genetic characteristics and could also be applied to studying the epidemiology and transmission of *T. crassiceps*. Moreover, as two of the most common molecular markers, they are flanked by regions of sequence conservation and are widely used in molecular ecology, population genetics and the diagnosis of parasitic organisms [40].

The partial *nad1* sequence (OM992098) retrieved in this study showed a 100% homology with *T. crassiceps* sequences detected in rodents: the steppe lemming (*Lagurus lagurus*) from Japan (LC644712.1) and the eastern meadow vole (*Microtus pennsylvanicus*) from the USA (EU544603.1); the definitive host, the red fox (*Vulpes vulpes*), from Croatia (KT757523.1); a sequence from Australia (AF216699.1). The partial *cox1* sequence retrieved in this study revealed a 100% homology with *T. crassiceps* sequences isolated from predators—the red fox (*Vulpes vulpes*) from Italy (MT806358.1), a dog (*Canis lupus f. familiaris*) from the Czech Republic (KY321219.1), rodents such as the common vole (*Microtus arvalis*) from Poland (MN514031.1), the Senegal bushbaby (*Galago senegalensis*) from the Czech Republic (KY321221.1), and a captive ring-tailed lemur (*Lemur catta*) from Bosnia and Herzegovina (KY883633.1). Our results are in line with those of Bajer et al. [37], who concluded, on the basis of the molecular characteristics of tapeworms derived from both intermediate and final hosts, that the same *T. crassiceps* genotype was present in both types of hosts. In comparison to the consensus sequences, no differences or mutations in either of the analyzed gene fragments (*nad1* and *cox1*) could be detected, confirming that these gene fragments are very conservative.

## 4. Materials and Methods

### 4.1. Case Description

The subject of the study was an adult male ring-tailed lemur (*Lemur catta*) born on April 3, 2009, at the Opole Zoo (Poland). The animal was kept with a group of other lemurs on an island surrounded by a water moat. The Zoological Garden is located on Bolko Island, the main part of which is a park with free-living animals, many of which are foxes and martens. In the middle of February 2021, a subcutaneous bulge of 4 cm in diameter was observed in the abovementioned individual in the area of the right thigh. It was a soft, fluffing change. No clinical signs or abnormal animal behavior were observed—appetite, thirst and condition were as usual. A fine needle puncture of the lesion was performed, and numerous leukocytes were found in the collected material, mostly in bloody, straw-colored content. After that, three intramuscular injections of amoxicillin (Clamoxyl LA, Zoetis, Warsaw, Poland) at a dose of 15 mg/kg of body weight (b. w.) were performed by the vet at 48-h intervals. Approximately 10 days after the lesion was observed, the persistent swelling turned into a stout consistency. Due to the lack of improvement, it was decided that it should be surgically removed.

### 4.2. Surgery and Sample Collection

The surgery was performed on March 1, 2021, during which the animal was administered ketamine hydrochloride (ketamine 10%, Biowet Puławy, Poland) at a dose of 10 mg/kg of b. w. (intramuscularly). During the procedure, inhalation anesthesia with 2% isoflurane was administered. A lesion of 4 cm in diameter, located subcutaneously in the area of the broad fascia tensioner muscle (*m. tensor fasciae latae*) and the biceps femoris muscle (*m. biceps femoris*), was removed. The entire lesion was submitted to the Regional Veterinary Laboratory in Opole for detailed examination. In the laboratory, cysts were microscopically examined to determine their morphological features.

After the surgical procedure, the animal received the following preparations for 3 days: meloxicam (Loxicon 5 mg/mL, Norbrook Laboratories Ltd., Newry, UK) at a dose of 0.3 mg/kg b.w. (subcutaneous injection) and albendazole (Zentel, GlaxoSmithKline, Dublin, Ireland) at a dose of 40 mg/kg of b. w. (per os). At the time of writing (10 June 2022), there have been no clinical changes in the abovementioned animal: behavior, condition, appetite and thirst are normal.

The material containing the cysticerci was collected, fixed in 70% ethanol and sent to the Department of Parasitology and Invasive Diseases at the National Veterinary Research Institute in Puławy. Subsequently, the obtained material was frozen at –20 °C in individual tubes for further molecular identification.

### 4.3. DNA Isolation and PCR Amplification

DNA was extracted from individual cysticerci using the QIAamp DNA Mini Kit (QIAGEN, Hilden, Germany) according to the kit manual. Isolated DNA was stored and frozen at −20 °C until further use.

Two PCRs were conducted to detect partial mitochondrial genes: *nad1* and *cox1* (popular genetic markers) of the *Taenia* sp. The obtained products were used for further phylogenetic analysis.

The first PCR employed in the present study amplified a partial sequence of NADH dehydrogenase subunit 1 (*nad1* fragment gene) using a protocol designed by Bowles and McManus [41] and modified by Dybicz et al. [42], which has been described previously [43] and is reported in Table 1. The PCR amplified a fragment to about 500 bp using the primers JB11 and JB12 (Table 1).

The second PCR employed in the present study amplified a partial sequence of cytochrome c oxidase subunit 1 (*cox1* fragment gene) using a protocol designed by Bowles et al. [44] and modified by Casulli et al. [45], which has been described previously [43] and is reported in Table 1. The PCR amplified a fragment to about 446 bp using primers CO1F and CO1R (Table 1).

For each PCR, a negative control (nuclease-free water) and positive controls (template DNA from *Taenia hydatigena*, *Echinococcus granulosus* and *Echinococcus multilocularis*) were included. The PCRs (*nad1* and *cox1*) were carried out in a TProfessional (Biomtera) thermocycler under conditions according to Table 2. The received PCR products were separated with horizontal electrophoresis in 2% agarose gels stained by SimplySafe stain (EURx, Gdańsk, Poland). The gels were visualized using the Fusion Fx, Fusion Capt Advance software supplied by Vilber Lourmat (Collégien, France).

## 5. Sequencing and Phylogenetic Analysis

The amplicons were selected for sequencing based on their molecular size. Sequencing was performed at a commercial company (Genomed S.A., Warsaw, Poland) using Sanger dideoxy sequencing. Phylogenetic analyses were conducted separately for each molecular marker *(nad1*, *cox1*). The forward and reverse sequences were analyzed, aligned and trimmed using the Geneious Alignment algorithm in the Geneious Prime bioinformatics software platform. The obtained consensus sequences were aligned with sequences from the GenBank Collection, using the BLAST nucleotide algorithm to confirm species identification and screen for the presence of single nucleotide polymorphisms (SNPs) with program settings in Geneious Prime. Furthermore, phylogenetic analysis was conducted using sequences available in GenBank as outgroups. A phenogram was created by applying the Tamura–Nei genetic distance model and the neighbor-joining building method with 1000 bootstrap replications in Geneious Prime. Nucleotide sequences obtained in this study were submitted to the GenBank database under the accession numbers OM992098 (*nad1*) and OM996999 (*cox1*).

## 6. Conclusions

To the best of our knowledge, this is the first report of *T. crassiceps* cysticercosis in a nonhuman primate in Poland. Although the source of infection and mode of transmission remain unclear, the fox population living in close proximity to the lemur enclosure is probably related to this infection. As mentioned earlier, nonhuman primates in zoos appear to be highly susceptible for *T. crassiceps* cysticercosis, which may be a sign of the zoonotic potential of other cestode parasite species. Our case report, along with scarce data about *T. crassiceps* in Poland, points to the necessity of testing domestic and wild carnivores, especially red foxes, for the presence of this parasite, which may be a zoonotic threat. Furthermore, our case additionally emphasizes the risk posed by these parasites and other parasitic diseases, especially zoonoses, when there is a probability of contact between zoos and wild animals.

## Figures and Tables

**Table 1:** Primer sequences for amplification of partial *nad1* and *cox1* genes.

Amplified Gene	Primer Name	Sequence (5′-3′)	Amplicon Size [bp]	References
*nad1*	JB11	AGATTCGTAAGGGGCCTAATA	~500	[41,42]
	JB12	ACCACTAACTAATTCACTTTC		
*cox1*	CO1F	TTTTTTGGCCATCCTGAGGTTTAT	~446	[44,45]
	CO1R	TAACGACATAACATAATGAAAATG		

**Table 2:** Thermocycler conditions.

Amplified Gene	Initial Denaturation	Number of Cycles	Denaturation	Annealing	Elongation	Final Extension Step
	Temp./Time [min]		Temp./Time [s]			Temp./Time [min]
*nad1*	95 °C/3 min	35	95 °C/60 s	50 °C/60 s	72 °C/60 s	72 °C/5 min
*cox1*	94 °C/7 min	38	94 °C/30 s	55 °C/30 s	72 °C/30 s	72 °C/5 min

## Data Availability

The partial nucleotide sequences of *Taenia crassiceps* mitochondrial cytochrome oxidase subunit 1 (*cox1*) gene and NADH dehydrogenase subunit 1 (*nad1*) gene were deposited in the NCBI database and are publicity available under the accession numbers OM996999 and OM992098, respectively.
